# Multi-parametric muscle and fat correlation of computed tomography parameters to outcomes in a total hip arthroplasty population

**DOI:** 10.1186/s12891-017-1926-1

**Published:** 2018-01-08

**Authors:** Michael A. Heffler, Ryan Barlow, Yin Xi, Daichi Hayashi, Hayden Box, Michael Huo, Avneesh Chhabra

**Affiliations:** 1 Department of Orthopedic Surgery, University of Texas Southwestern Medical Center, Dallas, TX USA; 2 Department of Radiology, University of Texas Southwestern Medical Center, 5323 Harry Hines Blvd, Dallas, TX 75390-8896 USA; 3 Department of Radiology, Boston University Medical Center, Boston, MA USA

**Keywords:** Computed tomography, Total hip arthroplasty, Segmentation, Harris hip score

## Abstract

**Background:**

Cross-sectional imaging is not currently used in planning Total Hip Arthroplasty (THA). The aim of our study is to determine correlations between CT parameters and outcomes following THA.

**Methods:**

A prospective registry of patients who underwent total joint arthroplasty was reviewed for patients who: (1) underwent THA, (2) had a CT between 1 year before and 6 months after surgery, and (3) completed perioperative WOMAC and Harris Hip Score (HHS) questionnaires. Two readers measured CT parameters, yielding mean Hounsfield Units, area, average diameter, and perimeter of the psoas major, gluteus medius and minimus muscles. A segmentation algorithm determined visceral and subcutaneous fat area, and waist circumference. ICC was calculated for each measurement to examine inter-reader agreement. Regression analyses were performed to select measurements with most impact on outcome scores.

**Results:**

Twenty-eight patients met inclusion criteria (17 female, 11 male), having mean (+/− standard deviation) age of 54.4 +/− 14.8 years and BMI 29.0 +/− 6.3 kg/m^2^. Correlations were found between HHS and age (0.650, *p* = 0.018), height (−1.263, *p* = 0.009), visceral-to-subcutaneous fat area ratio at the psoas level (0.511, *p* = 0.018), and waist circumference at the psoas level (1.759, *p* = 0.002). Inter-reader analysis showed ICC > 0.850 for all measurements.

**Conclusion:**

Age and height, as well as CT-derived visceral-to-subcutaneous fat area ratio and waist circumference significantly correlate with postsurgical HHS scores following THA. Our study suggests that parameters derived from cross-sectional CT imaging can be useful additional preoperative planning tool for THA.

## Background

Almost 700,000 Total Joint Arthroplasties (TJA) are performed in the United States each year, which include over 230,000 Total Hip Arthroplasties (THA) [1]. It is estimated that 15.1% of the THAs performed each year are revision procedures rather than primary operations, the economic impact of which is profound [1]. From 1997 to 2003, 19% of all Medicare spending on THA was consumed by revision operations [2]. THA is a widely used procedure, and the prevalence is projected to increase to as many as 668,700 procedures in 2030, with 96,700 (14.4%) of those estimated to be revision THA [1]. Methods to identify patients who will most likely benefit and likewise identify patients who will fail THA are immensely important because they can potentially aid in optimizing healthcare spending.

Cross-sectional imaging modalities, such as Magnetic Resonance Imaging (MRI) and Computed Tomography (CT), are not commonly used in preoperative screening of prospective THA patients. In 2015, Wiater et al. retrospectively examined a registry of patients who underwent reverse total shoulder arthroplasty (RTSA) and found significant correlations between radiographic parameters from MRI and postoperative outcome assessments [3].

CT measurement of the psoas major muscle has been used as a surrogate measure of sarcopenia [4]. It has been shown in some surgical and trauma populations that increased rates of overall morbidity [4–6], mortality [7], and decreased quality-of-life [8] significantly correlate with decreased psoas major area, volume, and density. The gluteal (abductor) muscles are considered highly important for overall hip function, especially for function after THA [9]. Due to superior soft tissue contrast and spatial resolution, CT examination of the pelvis can be used to evaluate both the abductor and psoas major muscle groups. In addition, evaluation of muscle size, muscle density, and fat segmentation with an analysis of fat fractions can be performed using independent software in a reproducible fashion.

Overall, there is a knowledge gap as to how these CT parameters ultimately can be related to outcomes of THA. No previous study has evaluated multi-compartment tissue analysis in the same setting and its relationship to outcomes of THA. The aim of our exploratory study was therefore to determine correlations between CT parameters and outcomes following THA.

## Methods

### Subjects

Patients were selected from a prospectively constructed registry of TJA patients at our institution. A retrospective review of this registry was performed under a HIPPA waiver and as part of an Institutional Review Board-approved study. This review yielded 802 TJA patients, 318 of whom underwent THA between 2006 and 2014.

At their initial visit, 6 months postoperatively, and 12 months postoperatively, THA patients completed the Western Ontario and McMaster Universities Osteoarthritis Index (WOMAC) Hip Score and Harris Hip Score (HHS) questionnaires in addition to normal follow-up.

We included patients who [1]: underwent THA [2], had a pelvic CT study performed for any reason in the time 1 year before or 6 months after the surgery, and [3] completed at least one set of postoperative questionnaires in addition to the initial visit questionnaires.

### Image analysis and reader training

All CT images were reconstructed as 3 mm slices in a soft tissue window for evaluation. Images were processed using an independent software program, Aquarius intuition (TeraRecon, Foster City, CA).

Two second-year medical students (MH, RB) served as readers, and independently measured parameters at two levels. Each reader was trained by a board-certified, fellowship trained radiologist (AC) to identify and analyze defined slices and measure selected regions of interest (ROIs) (See Slice and ROI Selection). This training consisted of two sessions, each two-hours in length, where the readers were taught to identify the defined slices and measure the selected ROIs on CT images from non-study patients. Readers then practiced this workflow under the supervision of the radiologist.

Additionally, 10% of the study sample (3 CTs) were used as a training set. These images were measured by the two readers using separate but identical workstations, working simultaneously and cooperatively to ensure a standardized workflow. Subsequently, the readers collected measurements using identical workstations, independent of one another on the remaining 90%, such that each CT was eventually measured twice, once by each reader.

### Slice and ROI selection

**Fig. 1:**
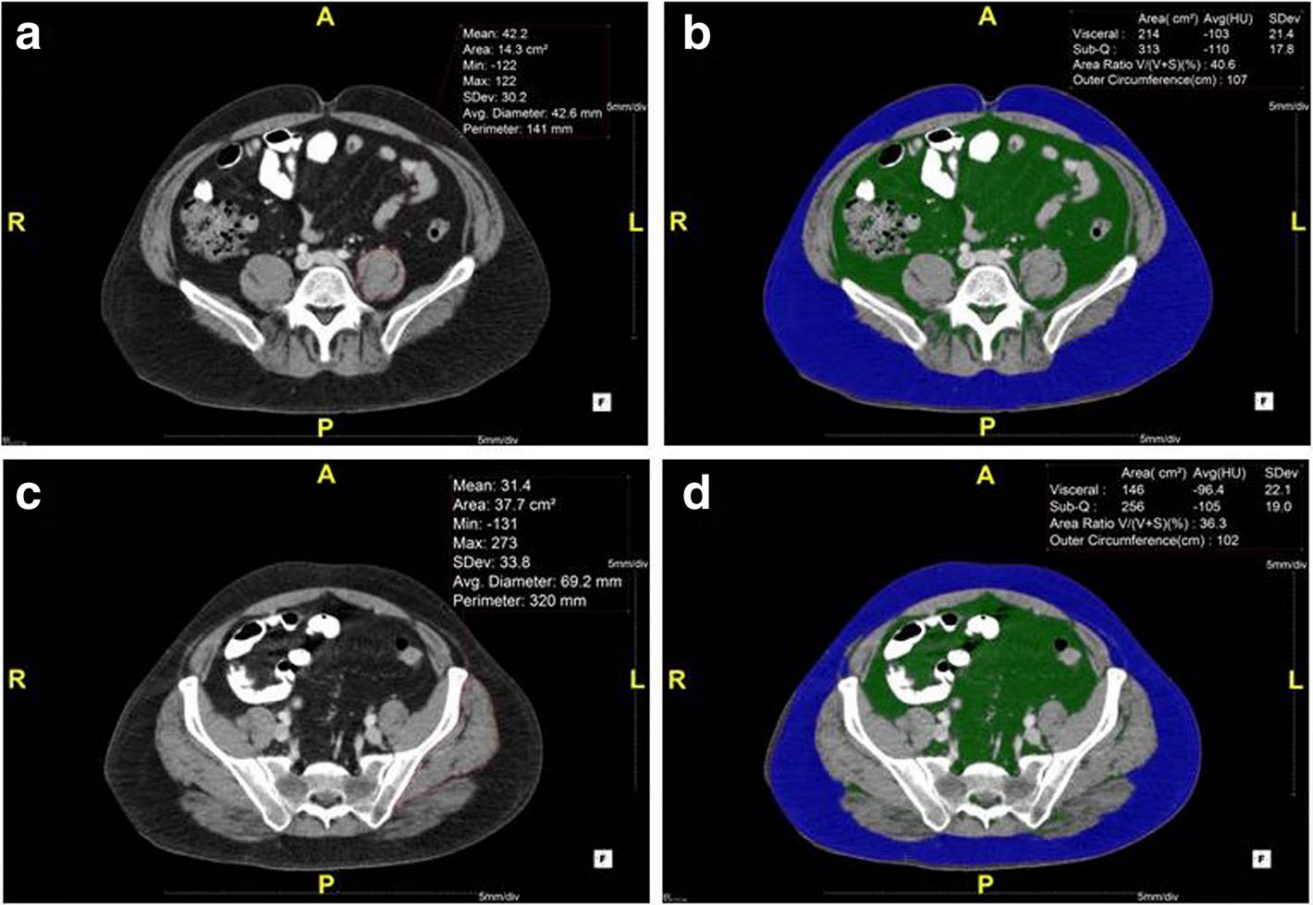
Representations of the CT slices analyzed: (**a**) psoas slice with muscle polygon, (**b**) psoas slice with fat analysis algorithm, (**c**) gluteal slice with muscle polygon, and (**d**) gluteal slice with fat analysis algorithm

Two slices of interest were defined, and on each slice ROIs were selected. The psoas slice was defined as the first axial slice superior to the sacroiliac joint, but which does not visualize the sacroiliac joint (Fig. 1a and b). The ROIs on the psoas slice included the psoas major muscles, both ipsilateral and contralateral to surgery. These muscles were selected as ROIs because cross-sectional psoas area measured from axial CT has been used as a marker for core muscle mass and sarcopenia, and has been shown to be correlated with postoperative functional outcomes after THA [4, 10]. The gluteal slice was defined as the axial slice demonstrating fully open S1 anterior foramina (Fig. 1c and d). The ROIs on the gluteal slice included the gluteus medius-minimus complex, again ipsilateral and contralateral to surgery. These muscles were identified as ROIs because they contribute to the overall musculature surrounding the hip and gluteus medius atrophy has been found to predict limping after THA at 6 months postoperatively [9, 11].

### Measurement of ROIs

On the psoas slice, three functions were performed: measurement of the psoas major ROI ipsilateral and contralateral to surgery and an automated fat-analysis segmentation algorithm. On the gluteal slice, three functions were performed: measurement of the gluteus medius-minimus ROI ipsilateral and contralateral to surgery and the same fat-analysis segmentation algorithm.

Measurement of each muscle was accomplished by manually constructing a polygon around the indicated muscle, yielding four parameters: mean Hounsfield units (Mean), cross-sectional area (Area), mean diameter (Diameter), and Circumference (Fig. 1a and c).

The fat-analysis segmentation algorithm also yielded four parameters: visceral fat area, subcutaneous fat area, ratio of visceral fat to total fat areas (Area Ratio, i.e. visceral fat area divided by the sum of visceral fat and subcutaneous fat areas), and the waist circumference of the patient (Outer Circumference) (Fig. 1b and d).

### Statistical analysis

A sensitivity analysis was performed between the study group and the overall THA group to determine whether the study sample differed significantly from the excluded registry population in demographic or clinical factors. Inter-reader agreement was assessed using the intraclass correlation coefficient (ICC) value for each measurement. Average measurements between the two readers were used for this analysis.

Additionally, univariate and multiple regression analyses were performed to determine the association between measurements and postsurgical outcome scores. The CT measurements and demographic data were used as independent variables and change in WOMAC and HHS were response variables. Change in WOMAC and HHS was defined as the difference between 12-month and initial scores.

In the multiple regression analysis, all CT measurements together with age, BMI, height, weight, and sex were initially included. A stepwise selection algorithm was then used to select the independent variables that most affected the response variables based on the F statistic. Using the selected independent variables, a multivariate model was constructed which was used to predict change in WOMAC and HHS.

All *p*-values of less than 0.05 were considered to be statistically significant. All statistical analysis was performed using Statistical Analysis Software (SAS Institute, Cary, NC).

## Results

### Subjects

**Fig. 2:**
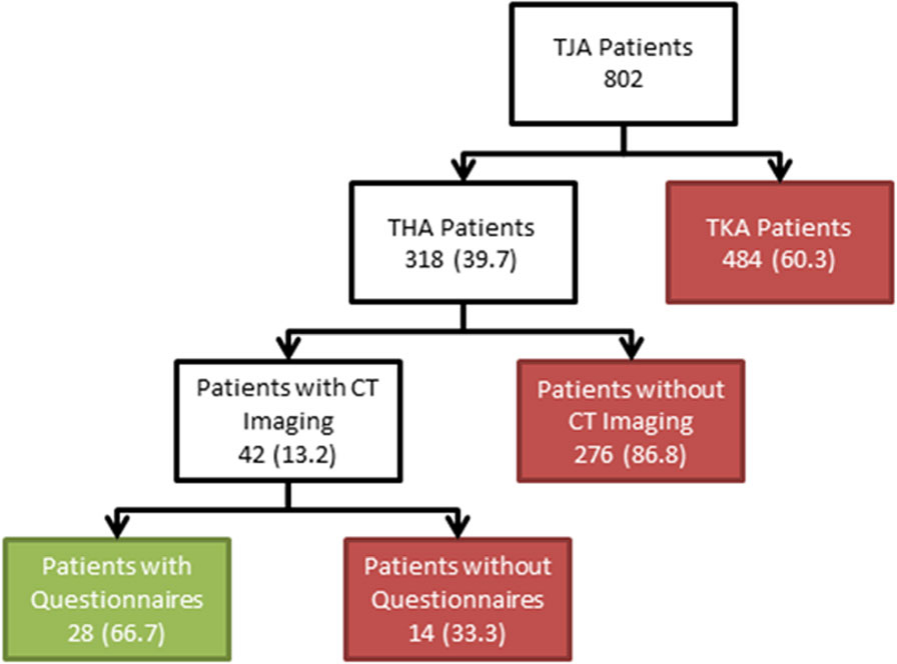
Flowchart depicting patient inclusion and exclusion

Of the 318 THA patients in the registry, 13.2% (42/318) had a CT study performed in the indicated time frame. Of those 42 patients who underwent CT imaging, 66.7% (28/42, 17 females, 11 males) had a complete set of postoperative questionnaires (Fig. 2). Mean (+/− standard deviation) age was 54.4 +/− 14.8 years, height was 165.9 +/− 9.0 cm, weight was 80.0 +/− 19.3 kg, and mean BMI was 29.0 +/− 6.3 kg/m^2^. Mean (+/− standard deviation) initial WOMAC score was 62.7 +/− 18.4 and initial HHS was 38.4 +/− 17.8.

### Statistical analysis

The study population was found to be similar to the overall THA group, with height being slightly different (Table 1). Analysis of agreement between the two readers showed an ICC > 0.85 for all measurements (Table 2). No significant correlation was found between any demographic or CT parameters and change in WOMAC Hip Score.

**Table 1:** Sensitivity analysis between the study group and overall registry

Parameter	Excluded (*n* = 290)	Included (*n* = 28)	*p*-value	Registry THA Patients (*N* = 318)
Weight (kg)	86.38 ± 19.95	80.00 ± 19.25	0.089	85.88 ± 19.65
Height (cm)	170.2 ± 11.4	165.92 ± 8.98	0.042	170.2 ± 11.1
BMI	29.69 ± 5.73	28.96 ± 6.29	0.588	30.07 ± 11.87
Age	57.20 ± 12.57	54.43 ± 14.76	0.167	58.80 ± 12.71
Initial WOMAC Hip Score	58.74 ± 20.87	62.71 ± 18.37	0.519	59.16 ± 20.63
12 Month WOMAC Hip Score	–	30.04 ± 24.75	–	–
WOMAC Hip Score Change	–	−31.74 ± 27.05	–	–
Initial Harris Hip Score	41.75 ± 15.66	38.36 ± 17.77	0.225	41.45 ± 15.86
12 Month Harris Hip Score	–	67.08 ± 21.89	–	–
Harris Hip Score Change	–	27.56 ± 23.53	–	–
Females	141	17	–	158
Males	149	11	–	160
Right Sided Surgery	141	12	–	153
Left Sided Surgery	134	16	–	150
Bilateral Surgery	15	0	–	15

**Table 2:** Results of inter-reader agreement analysis, with ICC shown for each measurement

Measurement	ICC Value	Measurement	ICC Value
Ipsilateral Iliopsoas Mean (HU)	0.978	Ipsilateral Gluteal Mean (HU)	0.914
Ipsilateral Iliopsoas Area (cm^2^)	0.990	Ipsilateral Gluteal Area (cm^2^)	0.895
Ipsilateral Iliopsoas Diameter (mm)	0.989	Ipsilateral Gluteal Diameter (mm)	0.905
Ipsilateral Iliopsoas Perimeter (mm)	0.946	Ipsilateral Gluteal Perimeter (mm)	0.957
Contralateral Iliopsoas Mean (HU)	0.959	Contralateral Gluteal Mean (HU)	0.991
Contralateral Iliopsoas Area (cm^2^)	0.992	Contralateral Gluteal Area (cm^2^)	0.930
Contralateral Iliopsoas Diameter (mm)	0.990	Contralateral Gluteal Diameter (mm)	0.940
Contralateral Iliopsoas Perimeter (mm)	0.858	Contralateral Gluteal Perimeter (mm)	0.959
Iliopsoas Visceral Fat Area (cm^2^)	0.987	Gluteal Visceral Fat Area (cm^2^)	0.980
Iliopsoas Subcutaneous Fat Area (cm^2^)	0.965	Gluteal Subcutaneous Fat Area (cm^2^)	0.976
Iliopsoas Area Ratio	0.904	Gluteal Area Ratio	0.921
Iliopsoas Outer Circumference (mm)	0.9995	Gluteal Outer Circumference (mm)	0.9999

**Fig. 3:**
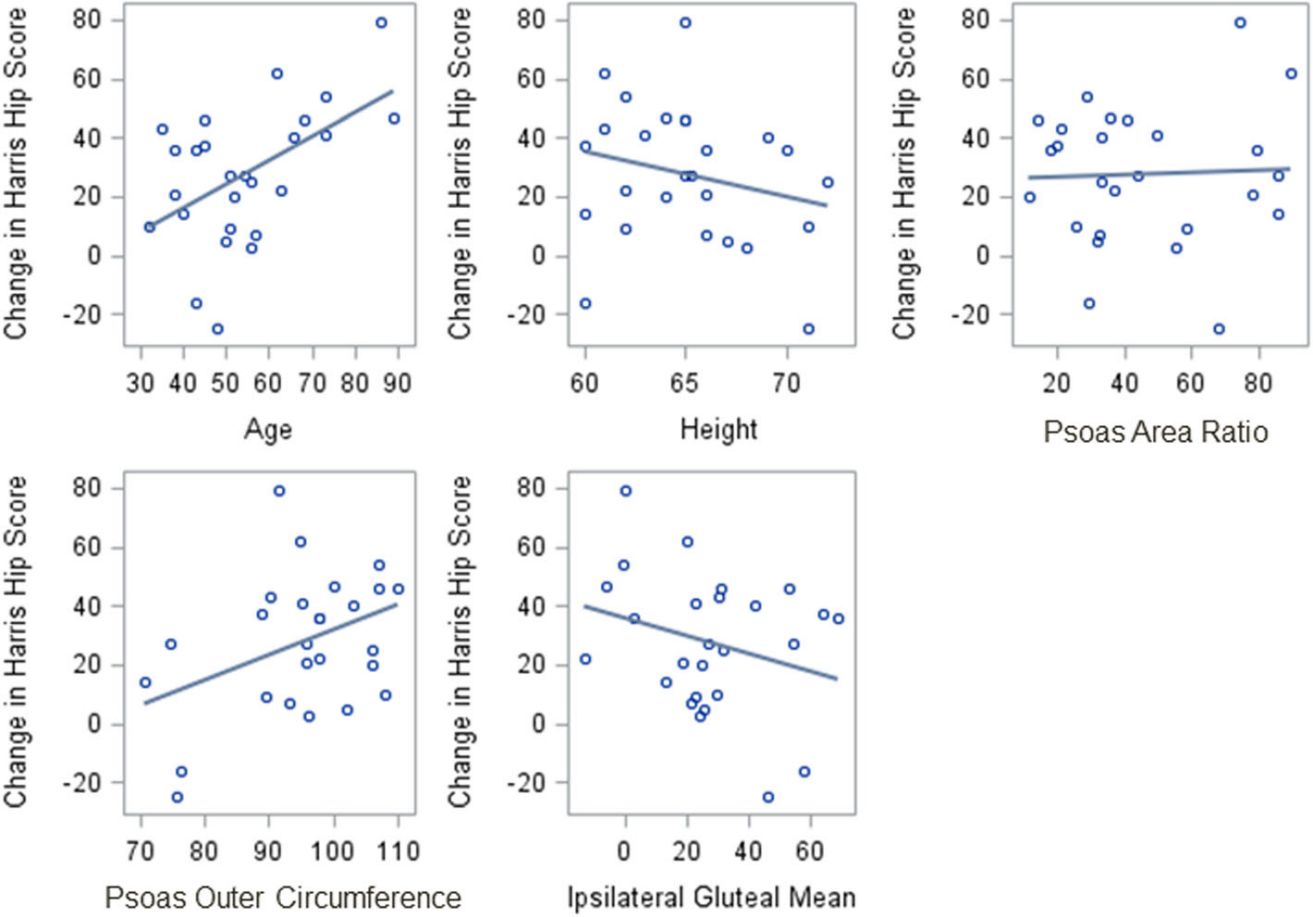
Scatterplots of significant univariate correlations between change in HHS vs. demographic and CT parameters. The line shown is the linear, univariate correlation

Significant univariate correlations were found between HHS and age (0.650, *p* = 0.018), height (−1.263, *p* = 0.009), Area Ratio at the psoas slice (Psoas Area Ratio, 0.511, *p* = 0.018), and Outer Circumference at the psoas slice (Psoas Outer Circumference, 1.759, *p* = 0.002) (Table 3, Fig. 3). Additionally, a non-significant trend was found between HHS and the mean Hounsfield units of the gluteus minimus-medius ipsilateral to surgery (Ipsilateral Gluteal Mean, 0.359, *p* = 0.108).

**Table 3:** Significant results of univariate correlation between change in HHS and demographic and CT parameters

Parameter	Correlation Coefficient	*p*-value
Age	0.65033	0.018
Height	−1.2628	0.009
Iliopsoas Area Ratio	0.51126	0.018
Iliopsoas Outer Circumference	1.75897	0.002
Ipsilateral Gluteal Mean	0.35905	0.108

Correlations are reported such that a one unit increase in a given CT measurement is associated with the reported increase in HHS. For example, an increase in height of 1 cm is associated with a 1.263 decrease in HHS, and a one Hounsfield unit increase in Ipsilateral Gluteal Mean is associated with a 0.359 increase in HHS.

**Fig. 4:**
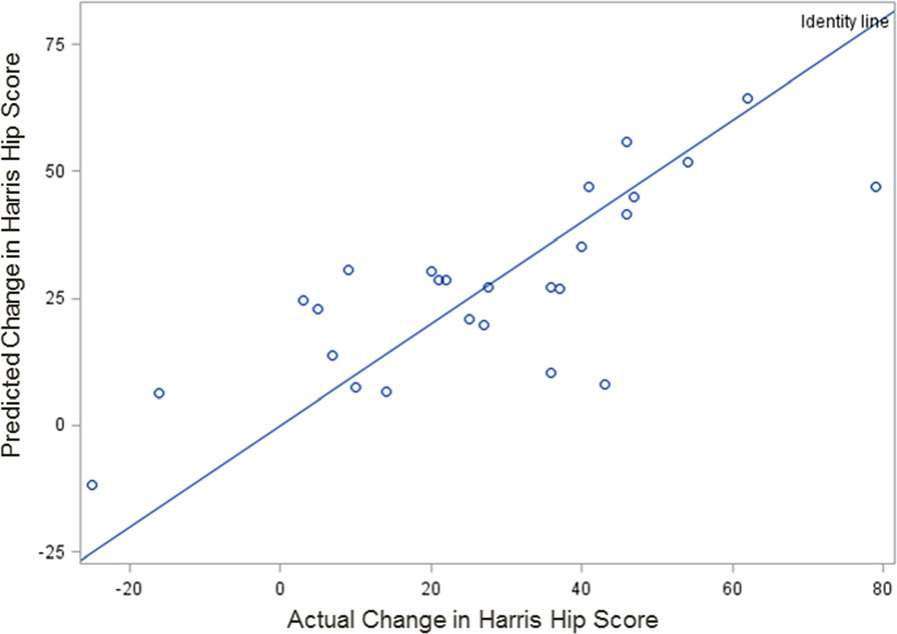
Scatterplot of change in HHS as predicted by the multivariate regression vs. actual change in HHS. The line shown is a linear regression with *R*^*2*^ = 0.5858

The resulting multivariate model that was produced was:

$$ Predicted Change=0.42773-1.263 Height+0.650 Age+0.511 PAR+1.759 POC+0.359 IGM $$

where PAR is Psoas Area Ratio, POC is Psoas Outer Circumference, and IGM is Ipsilateral Gluteal mean. When used to predict change in HHS for each patient, the model performed with *R*^*2*^ = 0.5858 (Fig. 4).

## Discussion

To the best of the authors’ knowledge, this is the first attempt at evaluation of pelvis CT muscle and fat parameters and characterization of their bearing on THA outcomes. The software proved to be reliable in deriving muscle area, density, and circumference from CT images as confirmed by the inter-observer performance. The fat ratios are easily evaluated, yielding reproducible data regarding subcutaneous and visceral fat proportions. Outer waist circumference was also reproducibly measured.

While no significant correlation was found between any CT measurement and change in WOMAC Hip Score, it is apparent that some CT parameters correlated with the postoperative HHS assessment tool. However, some of the correlations we observed are not what would be expected. For instance, larger waist circumferences and higher visceral fat fractions, surrogate markers of obesity, have been shown to be associated with negative surgical outcomes [12, 13], but in this study, they were found to be associated with improved outcome after THA. We believe this anomaly may be due to the limitations of this study, such as its retrospective nature and relatively small sample size. However, a sensitivity analysis showed that the data adequately represented the overall THA study group.

Our data reflects a preliminary exploration of this topic and future studies should include prospective enrollment and a standardized multivariate statistical approach including intra-reader performance. Quantitative information obtained from preoperative imaging not only holds diagnostic value, it may also aid in prognostication. This information may prove beneficial in preoperative patient counseling and might aid preoperative and postoperative decision-making by identifying subpopulations of patients who may benefit by therapy aimed at improving muscle properties before undergoing THA. A follow-up study should involve prospective recruitment of patients with focus on uniform imaging procedures and consistent collection of outcome data in order to ensure a large sample size. While MRI is also a potential technique for the above measurements, in this retrospective evaluation CTs were available for these patients who uniformly had hip scoring performed prospectively. In addition, the Aquarius software used in this study is more useful for segmentation using CT data. Although MRI can provide fat fraction analysis with recent advanced Dixon techniques, it happens to be a more expensive study and the imaging may be limited by metal artifacts due to hip prosthesis. There has been recent encouraging work on muscle and fat composition using MRI with qualitative and quantitative muscle and fat fraction analyses yielding meaningful correlations of muscle volume and intramuscular steatosis with muscle power and post-surgical outcomes [14–17]. Similar work could be potentially performed in THA population using metal reduction techniques. Lastly, volumetric multi-slice evaluation is now available with recent software upgrades. In a future study, it could be employed using a similar dataset to determine whether correlations are markedly different from those presented in this study.

## Conclusions

To conclude, multi-compartment tissue parameters derived from cross-sectional CT imaging can be useful as an additional preoperative planning tool for THA and establish a foundation for future prospective studies in this domain to expand validity of our preliminary results.

## Abbreviations

CT: Computed Tomography
HHS: Harris Hip Score
ICC: Intra-class Correlation Coefficient
MRI: Magnetic Resonance Imaging
THA: Total Hip Arthroplasty
TJA: Total Joint Arthroplasty
WOMAC: Western Ontario and McMaster Universities Osteoarthritis Index

## References

[1] Kurtz S, Ong K, Lau E, Mowat F, Halpern M (2007). Projections of primary and revision hip and knee arthroplasty in the United States from 2005 to 2030. J Bone Joint Surg Am.

[2] Ong KL, Mowat FS, Chan N, Lau E, Halpern MT, Kurtz SM (2006). Economic burden of revision hip and knee arthroplasty in Medicare enrollees. Clin Orthop Relat Res.

[3] Wiater BP, Koueiter DM, Maerz T (2015). Preoperative deltoid size and fatty infiltration of the deltoid and rotator cuff correlate to outcomes after reverse total shoulder arthroplasty. Clin Orthop Relat Res.

[4] Jones KI, Doleman B, Scott S, Lund JN, Williams JP (2015). Simple psoas cross-sectional area measurement is a quick and easy method to assess sarcopenia and predicts major surgical complications. Color Dis.

[5] Joglekar S, Asghar A, Mott SL (2015). Sarcopenia is an independent predictor of complications following pancreatectomy for adenocarcinoma. J Surg Oncol.

[6] Cornet M, Lim C, Salloum C (2015). Prognostic value of sarcopenia in liver surgery. J Visc Surg.

[7] Englesbe MJ, Patel SP, He K (2010). Sarcopenia and mortality after liver transplantation. J Am Coll Surg.

[8] Fairchild B, Webb TP, Xiang Q, Tarima S, Brasel KJ (2015). Sarcopenia and frailty in elderly trauma patients. World J Surg.

[9] Lachiewicz PF (2011). Abductor tendon tears of the hip: evaluation and management. J Am Acad Orthop Surg.

[10] Wakabayashi H, Watanabe N, Anraku M, Oritsu H, Shimizu Y (2016). Pre-operative psoas muscle mass and post-operative gait speed following total hip arthroplasty for osteoarthritis. J Cachexia Sarcopenia Muscle.

[11] Nankaku MPP, Tsuboyama TPM, Aoyama TPM, Kuroda YPM, Ikeguchi RPM, Matsuda SPM (2016). Preoperative gluteus medius muscle atrophy as a predictor of walking ability after total hip arthroplasty. Phys Ther Res.

[12] Kovesdy CP, Czira ME, Rudas A (2010). Body mass index, waist circumference and mortality in kidney transplant recipients. Am J Transplant.

[13] Aquina CT, Rickles AS, Probst CP, et al. Visceral obesity, not elevated BMI, is strongly associated with incisional hernia after colorectal surgery. Dis *Colon rectum* 2015;58:220-227. 10.1097/DCR.0000000000000261 25585081

[14] Marcon M, Berger N, Manoliu A (2016). Normative values for volume and fat content of the hip abductor muscles and their dependence on side, age and gender in a healthy population. Skelet Radiol.

[15] Lang T, Cauley JA, Tylavsky F (2010). Computed tomographic measurements of thigh muscle cross-sectional area and attenuation coefficient predict hip fracture: the health, aging, and body composition study. J Bone Miner Res.

[16] Inacio M, Ryan AS, Bair WN, Prettyman M, Beamer BA, Rogers MW (2014). Gluteal muscle composition differentiates fallers from non-fallers in community dwelling older adults. BMC Geriatr.

[17] Lee S, Lucas RM, Lansdown DA (2015). Magnetic resonance rotator cuff fat fraction and its relationship with tendon tear severity and subject characteristics. J Shoulder Elb Surg.

